# Nanoparticles for antiparasitic drug delivery

**DOI:** 10.1080/10717544.2019.1692968

**Published:** 2019-11-20

**Authors:** Yuzhu Sun, Dongmei Chen, Yuanhu Pan, Wei Qu, Haihong Hao, Xu Wang, Zhenli Liu, Shuyu Xie

**Affiliations:** a National Reference Laboratory of Veterinary Drug Residues (HZAU) and MAO Key Laboratory for Detection of Veterinary Drug Residues, Wuhan, China;; b MOA Laboratory for Risk Assessment of Quality and Safety of Livestock and Poultry Products, Huazhong Agricultural University, Wuhan, China

**Keywords:** Nanoparticles, antiparasitic drugs, bioavailability, transport, therapy effects

## Abstract

As an emerging novel drug carrier, nanoparticles provide a promising way for effective treatment of parasitic diseases by overcoming the shortcomings of low bioavailability, poor cellular permeability, nonspecific distribution and rapid elimination of antiparasitic drugs from the body. In recent years, some kinds of ideal nanocarriers have been developed for antiparasitic drug delivery. In this review, the progress of the enhanced antiparasitic effects of different nanoparticles payload and their influencing factors were firstly summarized. Secondly, the transport and disposition process in the body were reviewed. Finally, the challenges and prospects of nanoparticles for antiparasitic drug delivery were proposed. This review will help scholars to understand the development trend of nanoparticles in the treatment of parasitic diseases and explore strategies in the development of more efficient nanocarriers to overcome the difficulty in the treatment of parasite infections in the future.

## 1．Introduction

Parasites are a class of pathogens that are more harmful to human and livestock than bacteria, and they generally induce chronic diseases. Unlike most bacterial infectious diseases with rapid onset and obvious symptoms, many parasitic diseases are hardly diagnosed timely and thus bringing great economic losses to animal husbandry (Roberts et al., 1994). Moreover, many parasites are zoonotic pathogens that they can spread between humans and animals, thus posing huge risk to human health. For example, cystic echinococcosis (hydatid disease), a chronic helminthic disease, affects the human, domestic, and wild animals. This disease causes a reduction in the performance by 10% for the infected animals, through the decrease in the meat quality, milk production, and surviving of the offspring. Parasites always have distinct growth stages for surviving from one generation to the next. The parasites in different stages always produce distinct sensibility against the same antiparasitic drugs. Most parasites have life cycles containing intermediate organisms or vectors, which transport them from one to another host. Also, parasites can reside in host cells and establish reservoirs from which reinfection will occur, which often results in the long term and repeated infections. These properties lead to the considerable treatment difficulty for parasitic infections.

Chemical antiparasitic drugs are mainly used for controlling parasitic diseases. They are critical in animal husbandry development and animal health safety, but most antiparasitic drugs have low bioavailability due to their insolubility and their short half-life. Therefore, the treatment of parasitic diseases needs frequent dosage for a long-time because of the long-life cycles of parasites. The repeated treatment might cause animal stress, big labor intensity of farmer and drug resistance (Vercruysse et al., 2007). For example, praziquantel is hardly soluble in aqueous solution and its bioavailability is poor regarding its natural metabolism in the liver and rapid elimination from the body. The repeated high doses for a long time are required in the treatment of cestode infection and thus might result in dizziness, tiredness, nausea, and hangover sense.

To avoid these limitations, novel approaches are required for enhancing the effects of antiparasitic drugs. With the rapid development of nanomedicine and people’s increasing requirements for the treatment of parasitic diseases, nanoparticles, especially organic nanoparticles, have attracted people’s attention for antiparasitic drug delivery. The organic nanocarriers are usually made of natural or synthetic polymers, solid lipids at room temperature, phospholipids, and cholesterol. These materials were prepared into particles in size ranges between 10 and 1000 nm and thus obtain some outstanding properties due to their substantial specific surface area and strong adhesion (Wagner et al., 2006). Different nanocarriers containing ‘solid lipid nanoparticles (SLNs), polymeric nanosystems (i.e. polymeric nanospheres, nanoparticles and micelles), nanocrystals, and liposomes’ have been attracted people’s attention for delivery of anti-parasitic drugs. The antiparasitic drugs are loaded into the nanoparticles physically or chemically through adsorption, encapsulation and conjugation. The payload can be released through desorption, dissolution, and degradation. These nanoparticles can be administered by oral, intragastric, duodenum, skin, pulmonary, intravenous, and other routs according to the requirements of disease treatment and drug properties (Zhang et al., 2012; Hamori et al., 2014; Chen et al., 2015; Xu et al., 2017). These nanoparticles can penetrate the biological barriers, protect drug degradation from enzymes, and hold satisfactory targeting, physical stability, controlled release, and effective intracellular delivery and accumulation, etc. (Das & Chaudhury, 2011; Negi et al., 2013). For example, our groups demonstrated that hydrogenated castor oil SLNs increased the bioavailability and the mean residence time (MRT) of praziquantel in dogs by 5.67 and 4.94 folds, respectively (Xie et al., 2010). The therapeutic efficacy of the SLN suspension against tapeworm in diseased dogs was enhanced by a single subcutaneous dose (Xie et al., 2011). At present, nanoparticles have shown broad development prospects in the application of antiparasitic drug delivery.

In this review, we searched PubMed, Scopus, Web of Science, and Cochrane Central register of related publications about the application of nanoparticles in the treatment of parasitic infection using relevant keywords ((nanoparticles or polymer nanoparticles or solid lipid nanoparticle or liposomes or nanocrystal) and (antiparasitic drug or parasitic infection)). There are about 6000 records and 150 of closely related paper were screened for eligible studies. Based on these related publications, we systematically discuss the nanoparticles progress, challenges, and perspectives in the delivery of antiparasitic drugs to discover new trends in the expansion of more effective nanocarriers to overwhelm the difficulty in parasitic diseases therapy.

## 2. Problems of antiparasitic drugs in the treatment of parasitic diseases

Antiparasitic drugs are still the best choice for the control of animal parasitic diseases. In the veterinary clinic, the antiparasitic drug formulations mainly include common tablets, powders, and injections. Most of these conventional formulations are poorly absorbed due to their insolubility and discharged with excrement. For example, most members of benzimidazoles are hardly absorbed in the body because of their poor solubility and stability in the gastrointestinal tract. Some antiparasitic drugs, e.g. ivermectin and praziquantel, are susceptible to enzymatic degradation or inactivation in animals and thus exhibiting strong first-pass effects. They also show poor penetration across the biological membrane barriers of tissues and cells, which also result in reduced bioavailability and hardly achieve the expected therapeutic effect (Lu et al., 2017; Babita et al., 2018). In addition, most parasites, e.g. Leishmania amphotericin B, reside in intracellular, which leads to poor therapeutic effects due to the weak transmembrane and intracellular transfer ability of antiparasitic drugs (Silva et al., 2016). It is also reported that the resistances of antiparasitic drugs are becoming more and more serious due to the vast and irrational usages. A large and multiple dose often need to obtain satisfactory effects and are prone to produce protozoal resistance and toxic side effects (Balaña-Fouce et al., 1998).

## 3. Enhanced therapy effects of antiparasitic drugs by nanoparticles

With the continuous development and innovation of nanomedicine, nanoparticles have been researched for antiparasitic drug delivery to improve their bioavailability, sustained release, and intracellular penetrability performances. Immobilization of antiparasitic drugs on or into nanoparticles is an effective way to improve efficacy and decrease the toxic side effects of drugs. At present, some nanoparticles including liposomes, polymer nanoparticles, SLNs, nanosuspensions, and others, have been begun to study for antiparasitic drug delivery. This section focusses on the progress in the application of some main nanoparticles for antiparasitic drug delivery and their improved therapeutic effects (Table 1).

**Table 1.** Main nanoparticle delivery systems for antiparasitic drugs.

Drug delivery system	Advantages	Drug	Parasite	Technology	Effect	Ref
Liposomes	Targeting Excellent safety	Avermectin	/	/	Effect time *in vivo* extended to 30 days	Sun et al., 2014
		Avermectin	Swine fever	Rapid evaporation method	Significantly improved cure rate	Panwar et al., 2010
		Ivermectin	/	Rapid evaporation method	Extend action time	She et al., 2010
		Albendazole	Metchstodes multilocularis	/	Enhanced therapy effects	Wen et al., 1996
		Albendazole	/	/	35% reduction in release rate within 4 hours	Dvoroznakova et al., 2004
		Albendazole	Echinococcus multilocularis	/	Stimulated macrophage function and increased deworming efficacy	Liu et al., 2000
		Fenbendazole	Toxocarosis	/	Enhance drug efficacy	Velebný et al., 2000
		Praziquantel	Schistosomiasis	Rapid evaporation method	Improve anti-schistosomiasis activity	Mourão et al., 2005
		Praziquantel	Schistosomiasis	/	Half-life was extended by 5 times	Zhang et al., 2000
		Praziquantel	/	/	Half-life was extended by 5 times	Shaik et al., 2004
		Monensin	Plasmodium	Evaporation	Improved treatment effects	Rajendran et al., 2016
		Monensin	/	/	Enhanced effects against resistant parasites	Mukherjee et al., 2004
		Amphotericin B	Leishmaniasis	/	Reduced side effects	Balaña-Fouce et al., 1998
		Amphotericin B	Leishmaniasis	Cast film method	Targeted macrophages and avoided toxicity organs	Rathore et al., 2011
		Curcuminoids	Leishmaniasis	Thin-film hydration method	Avoided cardiotoxicity and hepatotoxicity	Aditya et al., 2012
		Curcuminoids	Plasmodium	Solvent diffusion–evaporation method	Improved bioavailability and reduced hemolysis rate	Ahmadnia et al., 2013
		Artemether	Plasmodium	Dimerization and self-assembly	Improved antimalarial activity and avoid hemolysis	Ismail et al., 2018
		Amphotericin B and allopurinol	Leishmaniasis	/	Showed nontoxicity and speeded up recovery	Khodabandeh et al., 2019
		Meglumine Antimoniate	Cutaneous Leishmaniasis	Fusion method	Increased penetration rate by approximately 60%	Moosavian et al., 2019
		Ivermectin	/	/	Avoiding the macrophage uptake	Gamboa et al., 2016
Solid lipid nanoparticles	Low toxicity Good biocompatibility Sustained release performance	Ivermectin	/	Ultrasonic crushing method	Slow release, enhanced transdermal effect	Dou, 2016
		Albendazole sulfoxide	/	High pressure homogenization	Improved drug activity	de Souza et al., 2014
		Albendazole	Bow locust	High shear homogenization and probe sonication methods	Extended release and increased efficacy	Marslin et al., 2017
		Praziquantel	Schistosomiasis	Hot homogenization and ultrasonication method	Increasing oral bioavailability by 14.9 times and extending *in vivo* circulation time to 88.3 hours	Xie et al., 2010
		Arteether	Plasmodium	High pressure homogenization	Avoiding gastric acid degradation and improving oral bioavailability	Dwivedi et al., 2014
		Praziquantel	Schistosomiasis	Ultrasonication method	Reduced cytotoxicity	Yang et al., 2009
		Praziquantel	Tapeworm	High pressure homogenization	Increasing bioavailability by 5.67 times and extending *in vivo* circulation time to 224.67 hours	Pensel et al., 2015
		Praziquantel	/	High-shear homogenization	Enhancing oral bioavailability by two times	Souza et al., 2012
		Praziquantel	Schistosomiasis	Solvent diffusion method	Improving bioavailability and reducing toxicity	Silva et al., 2016
		Praziquantel	Murine S. mansoni	/	Enhancing AUC0-24 by 8–9 times	Radwan et al., 2019
		Albendazole	/	High shear homogenization and probe sonication methods	Decreased toxicity to U-87 MG cells by 2.9 times	Marslin et al., 2017
		Paromomycin	Leishmania	High shear homogenization microemulsion technique	Improving the effectiveness of PM in killing the parasite and switching towards Th1 response.	Heidari-Kharaji et al., 2016
		Paromomycin	Leishmaniasis	High shear homogenization microemulsion technique	Inhibiting the parasite propagation and switching towards Th1 response	Heidari-Kharaji et al., 2016
		Dihydroartemisinin	Plasmodium	single-emulsion solvent evaporation techniques	Enhancing efficacy by 24% and 97.24% against chemosuppression at 2 mg/kg/d	Omwoyo et al., 2016
		Amphotericin B	Visceral leishmaniasis	Probe sonication-assisted nanoprecipitation technique	Enhancing bioavailability by 1.05-fold	Chaudhari et al., 2016
		Paromomycin	Leishmaniasis	High shear homogenization microemulsion technique	Enhancing effects	Kharaji et al., 2016
Nanosuspension	Simple preparationHigh drug loading Easy to expand production	Ivermectin	/	High pressure homogenization	Enhancing dissolution rate by 4 times	Starkloff et al., 2016
		Albendazole	/	High pressure homogenization	Increasing bioavailability by 2.96 times	Mittapalli et al., 2007
		Cyadox	/	Acid–base neutralization and high pressure homogenization	Increasing bioavailability by 359.1%	Sattar et al., 2017
		Aphidicolin	Leishmaniasis	/	Enhancing targeting	Kayser, 2000
		Bupravaquone	Cryptosporidium parvum	High pressure homogenization	Enhanced mucosal adsorption and targeting	Lemke et al., 2010
		Praziquantel	Taenia crassiceps cysticerci	/	Elevated anaerobic glycolytic activity against T. crassiceps cysticerci and enhanced insecticidal activity	Silva et al., 2016
		Amphotericin B	/	High pressure homogenization method	Enhanced solubility and bioavailability	Zhou et al., 2018
		Albendazole	/	Surfactant assisted media milling method	Increased solubility and dissolution rate	Fülöp et al., 2018
		Albendazole	Fox tapeworm Echinococcus multilocularis	High pressure homogenization	Reduced weight of the cysts by 77%	Pensel et al., 2018
		Usnic acid	/	The wet milling method	Enhanced C_max_ and AUC by 348% and 181%	Qu et al., 2018
		Artemether	Plasmodium	Wet milling technology	Parasitic rate reduced by 89%	Shah et al., 2016
Polymeric nanoparticles	Sustained release performance Targeting Good stability	Amphotericin B	Leishmaniasis	/	Enhanced effectiveness of deworming by twice	Lala and Basu, 2004
		Nifurtimox	Trypanosoma cruzi	Emulsion polymerization	Enhanced effectiveness and reduced parasitic rate by 87–94%	Gonzalezmartin et al., 2011
		Betulinic acid	Leishmania	Novel solvent and phase separation method	Improved drug efficiency and reduced side effects	Tahereh et al., 2018
		Paromomycin	Leishmaniasis	Ionic gelation method	Enhanced effects against the amastigote and reduced toxicity	Esfandiari et al., 2019
		Spiramycin	Toxoplasmosis	Ionotropic gelation method	90% reduction in parasitic rate	Hagras et al., 2019
		Chitosan	Cryptosporidium parvum oocysts	Ionotropic gelation method	Reduced the number of Cryptosporidium	Ahmed et al., 2019
		Spiramycin	Toxoplasmosis	Ionotropic gelation method	Reduced toxicity and enhanced insect resistance	Etewa et al., 2018
		Isoniazid	Tuberculosis	Spray-drying technique	Decreased cytotoxicity and enhancedinternalation in A549 cells.	Manca et al., 2013
		Clofazimine	Cryptosporidiosis	The flash nanoprecipitation	Increased solubility by 90 times	Zhang et al., 2017
		Nigella sativa oil	Leishmania infantum	/	Inhibiting up to 90% of parasites	Abamor et al., 2018
		Betulinic Acid	Leishmaniasis	Emulsion solvent evaporation technique	Enhanced anti-leishmanial activity.	Halder et al., 2018
		β-lapachone	Leishmaniasis	/	Reduced inflammation	Moreno et al., 2015
		Betulinic acid	Leishmaniasis	Drug adsorption and phase separation methods	Deworming rate was increased by 86%	Zadeh Mehrizi et al., 2018
		Polymyxin B	Leishmaniasis	Emulsion polymerization method	Macrophage targeting	Souza Ribeiro Costa et al., 2019
		Amphotericin B	Balamuthia mandrillaris	/	Enhanced targeting delivery and reduced toxicity	Kumar et al., 2017
		Paromomycin	Visceral leishmaniasis	Osmosis-based methodology	Parasitic rate was reduced by 3.6 times	Hönn and Göz, 2006

### 3.1. Liposomes

Liposomes are closed vesicles comprised of one or more lipid bilayers containing drugs in the bilayer and inner. Liposomes were firstly discovered and named by Bangham et al., 1965, and then firstly developed as a drug carrier by Gregoriadis et al. in the 1970s (Gregoriadis et al., 1974). As nanocarriers, liposomes have the advantages of targeting, controlling release and reducing toxicity. The latest report on the treatment of resistant visceral leishmaniasis with interferon gamma in combination with liposomal amphotericin B and allopurinol had no side effects and accelerated recovery (Khodabandeh et al., 2019). This report describes the first case of visceral leishmaniasis resistant to pentavalent antimonials and also the first use of combinational therapy in Iran. In recent years, it has been gradually applied to antiparasitic drugs. For example, the liposomal praziquantel and avermectin were reported to show better deworming effects (Mourão et al., 2005; She et al., 2010).

Liposomes can be targeted to specific tissues via controlling their self-specific properties or by attachment of specific ligands onto their surfaces. For example, praziquantel liposomes are mainly distributed in the liver and spleen that is rich in the reticuloendothelial system after intravenous injection thus obtaining more effective insecticidal effects since liver and spleen are the main parasitic sites of schistosomiasis (Zhang et al., 2000). The amphotericin liposomes with mannitol mainly distributed in the liver and spleen where the pathogen resides. Liposomal fenbendazole with glucan mainly concentrated in muscle and was more abundant than the plain liposomes with positive and neutral surface (Velebný et al., 2000). Leishmania is an intracellular parasite, while most antiparasitic drugs are hardly to enter the cells. Therefore, it is still difficult for Leishmania control. The mannosylated liposomes could more effective attack visceral Leishmania directly (Rathore et al., 2011), and in the study of Khodabandeh et al. (2019) and Moosavian et al. (2019), liposome also increased the repellent activity and reduced toxicity. It is more interesting that the phospholipid of liposomes can directly affect the parasite. The high concentration of lipid of liposomes without the drug can directly act on the parasites when it enters the cell and causes alteration in the motility and aspect of S. mansoni (Zhang et al., 2000). Some liposomes could be prepared by combining some chemically and biologically inert synthetic polymers to produce long-circulating liposomes (Asthana et al., 2015), furtherly prolonging the drug circulation time *in vivo*, thereby enhancing the effectiveness. For example, the duration of efficacy of liposomal avermectin was increased from 21 to 30 d (Sun et al., 2014). Liposomes were also reported to decrease the resistance occurrence of monensin (Rajendran et al., 2016). Also, the structures of some liposomes are like biomembranes, e.g. ivermectin liposomes constituents (soy lecithin and cholesterol) (Velebný et al., 2000), phospholipids in albendazole liposomes (Wen et al., 1996), phosphatidyliner in praziquantel liposomes (Zhang et al., 2000) and mannitol (Rathore et al., 2011) in amphotericin liposomes. These constituents can be biodegraded *in vivo* without producing any toxic substances, and simultaneously reduce the side effects of drugs (Balaña-Fouce et al., 1998; She et al., 2010; Rathore et al., 2011; Silva et al., 2016).

In all, liposomes have advantages of improved specific distribution, prolonged circulation, decreased toxicity, and fewer side effects of antiparasitic drugs. Their efficacy will be further refined and enhanced through surface modification by conjugating with proper moieties.

### 3.2. Solid lipid nanoparticles (SLNs)

Solid lipid nanoparticles (SLNs), originally proposed by Müller et al. in 1991, is a novel nanoscale delivery system of drugs that have been developed rapidly in recent years. It mainly uses natural or synthetic lipids as materials to adsorb, encapsulate or disperse drugs (Müller et al., 2000). The SLNs combine the advantages of classic oil-in-water emulsions, liposomes and polymer nanoparticles, such as easy mass-production and well physiological compatibility and degradability (Dingler et al., 1999). In the last year, our group has established the large-scale production technology for two SLNs formulation, which will promote the efficient process of SLNs.

As a relatively new and promising pharmaceutical formulation, it holds the advantage of increasing drug solubility, improving bioavailability and prolonging release. Some antiparasitic drug loaded SLNs have been developed in recent years. Praziquantel-loaded hydrogenated castor oil SLNs developed by our group only released 62.24% of the drug within 7 d and greatly improved the oral bioavailability and circulation time of praziquantel in mice and dogs (Xie et al., 2010; Xie et al., 2011). This might be due to that SLNs could adhere to the gastrointestinal mucosa and increase the mucosal permeability after oral administration due to their tiny sizes. The high surface area also improves the dissolution rate of insoluble praziquantel. Compared with the commercially available transdermal agent, ivermectin-loaded SLNs formulated by Dou et al., reduced the drug release by 40% within 48 h without burst release (Dou, 2016). *In vitro* release of albendazole from SLNs showed an extended-release profile (Marslin et al., 2017). Arteether (ART)-loaded SLNs showed slow and continuous release (Dwivedi et al., 2014). These effects might be due to avoiding acid degradation of ART in the stomach and thus improved its oral bioavailability and sustained release performance. Moreover, the release of the encapsulated drugs from SLNs could be modified by changing the kinds of lipids. For example, albendazole-loaded Compritol 888 ATO SLNs released 10.66 ± 1.7% drug *in vitro* within 24 h and was released slower than those of albendazole-loaded glyceryl trimyristate SLNs, indicating glyceryl trimyristate SLNs exhibits better-sustained release properties (Anjali et al., 2017; Marslin et al., 2017). Concerning the parasite diseases such as cystic echinococcosis, the improved bioavailability and sustained residence *in vivo* are always associated with increased clinical therapy efficiency. For example, our previous work showed hydrogenated castor oil SLNs significantly increased the bioavailability and MRT of praziquantel by subcutaneous routes and thus obtaining improved treatment effects against tapeworm in dogs (Xie et al., 2011). Albendazole-loaded solid lipid nanocapsules prepared with tricaprylin and caprylic-capric acid triglycerides at a ratio of 1:1 were reported to improve the bioavailability and cysts of infected mice and thus showed a better chemoprophylactic efficiency than albendazole suspension after oral delivery as 4 out of the 10 nanocapsules treated mice without any cysts, while the infection developed in all mice in the group of albendazole suspension (Pensel et al., 2015; Ullio Gamboa et al., 2019). It is also reported that SLNs can achieve dynamic effects for the intracellular parasites (e.g. Plasmodium and Leishman), which shows better deworming efficacy (Chaudhari et al., 2016; Heidari-Kharaji et al., 2016; Heidari-Kharaji et al., 2016; Kharaji et al., 2016; Omwoyo et al., 2016). In the study of Omwoyo et al., it even shows an increase of 97.4% in deworming rate (Omwoyo et al., 2016). The SLNs could enter the cell due to their small size of the particles. The lipid is decomposed by lysosomes because of their physiological compatibility and degradability when entering the cell, and then the payload could be swiftly released and directly affect the intracellular parasites.

In summary, although their antiparasitic effects have not been broadly researched, SLNs have emerged as promising alternatives to some other nanoparticles for enhancing the therapeutic action of antiparasitic drugs by a tunable release rate and specific targeting.

### 3.3. Nanosuspensions

The nanosuspensions consists of drug nanocrystals and a little of surfactants on its surfaces and usually exists as an aqueous dispersion (Müller et al., 2001). The highlight advantages of nanosuspensions are to augment the solubility, dissolution percentage and rate, and absorption of drugs (Sattar et al., 2017). It will be an ideal option when the principal difficulty of drug absorption is because of its poor solubility and dissolution velocity. It should be noted that the nanosuspensions are easily commercialized due to their low cost, high loading capability of drug with, easyscale-up production, and low or not any side effects. Currently, there are some nanocrystal products in medicine after the Rapamune^®^ firstly entered the market in 2000, while the anti-parasitic formulations based on nanocrystal have not yet come into the market.

Some antiparasitic drugs have been produced into nanosuspension with some prominent properties. The ivermectin nanosuspensions development by Starkloff et al. (2016) has a solubility four times larger than that of ivermectin alone due to the presence of nanoparticles and amorphous. A recent report found that ellagic acid nanoparticles prepared by antisolvent precipitation showed improved/sustained antibabesial effects in different cells and in the animal. The IC_50_ of ellagic acid nanoparticles for ‘*B. bovis*, *B. bigemina*, *B. divergens*, *B. caballi* and *T. equi*’ were ‘4.2, 9.6, 2.6 , 0.92 and 7.3 µM’, respectively, while the IC_50_ values of ellagic acid on ‘*B. bovis*, *B. bigemina*, *B. divergens*, *B. caballi* and *T. equi*’ were ‘9.58 , 7.87 , 5.41, 3.29 and 7.46 µM’, respectively (Beshbishy et al., 2019). Our groups have developed some nanosuspensions for albendazole, fenbendazole, and oxfendazole, which significantly increased their solubility, bioavailability, and peak serum drug concentrations. The bioavailability of albendazole nanosuspensions prepared by Kumar P (Mittapalli et al., 2007) is also increased by 2.14–2.96 times. When buparvaquone was administered in the form of nanosuspensions in the treatment of Cryptosporidium, many nanoparticles were found to adhere to the mucosa, and thus prolonging the residence time in the gastrointestinal tract, increasing bioavailability and simultaneous reducing the dosage and side effects of the drug (Kayser, 2001). As described in other granular pharmaceutical formulations, nanosuspensions are generally used to target phagocytic cells, but can also be delivered to specific sites such as central nervous system, spleen, liver, lung, and bone marrow depending on their particle characters and particular surfactant coating. Nanosuspensions of amphotericin B coated with polysorbate 80 and sodium cholate significantly increased the brain delivery and exhibited enhanced parasite inhibition *in vitro* (Lemke et al., 2010). Kayser (2000) prepared aphidicolin-nanosuspensions that can passively target macrophages via directly phagocytose by macrophages. Compared to dimethyl sulfoxide-dissolved drugs, nanosuspensions show increased activity against Leishmania about 140 times, indicating that the cellular uptake of nanoparticles is critical to improve the activity of their payload.

### 3.4. Polymer-based nanoparticles

Polymeric nanoparticles are a type of nanosized drug delivery system comprise of natural or synthetic polymers. Drugs could be entrapped, encapsulated, dissolved, or attached to the polymeric matrix. Polymeric nanoparticles could be divided into nanoparticles, nanospheres, or nanocapsules basing on preparation method and structure. Nanoparticles are the particles in size within 10–1000 nm with that the drug molecules are evenly distributed throughout the matrix materials. Nanocapsules are vesicular carriers where the drug is kept in a cavity bounded by a polymer membrane, while nanospheres are matrix systems where the drug molecules are evenly dispersed. In past years, polymeric nanoparticles have substantial expectations as a drug delivery carrier because of their ability for controlled release, targeting to organs/tissues and delivering different drugs such as proteins, peptides, and genes.

Recently, polymer nanoparticles have been explored to deliver the drugs for the anti-intracellular parasites, e.g. amphotericin B against Leishmania (Asthana et al., 2015; Kumar et al., 2017), and chloroquine and artemisinin against intracellular Plasmodium (Talisuna et al., 2004; Afonso et al., 2006; Tripathy et al., 2013). Nanoparticles have critical role in improving cellular penetration, intracellular retention and specific subcellular target, and even escape from intracellular enzymatic inactivation of drugs. For example, paromomycin-loaded mannosylated chitosan nanoparticles using dextran increased the amount across THP-1 cell after incubation of 6 h by 2.8–3.9 folds compared to non-mannosylated chitosan nanoparticles. The effect of paromomycin-loaded chitosan nanoparticles was more salient on amastigotes, while paromomycin-loaded mannosylated chitosan nanoparticles effectively affected both stages of the parasite, especially the amastigote (Esfandiari et al., 2019). The developed amphotericin B-loaded peptide (glycine) coated iron oxide nanoparticles (Fe nanoparticles (GINPs) by Kumar et al. (2017) et al. significantly increased the bioavailability and reduced off-target delivery of amphotericin B in body, and showed doubled effects against visceral leishmaniasis than that of free amphotericin B. Some groups have prepared protolith nanoparticles, with the goal of targeting the liver (Labhasetwar & Dorle, 1990; Mbela et al., 1992). Due to their strong targeting of polymer-based nanoparticles via modification, they can can reduce the toxicity of drugs to other untargeted organs. The neptochrome-loaded adhesive cyanoacrylate nanoparticles prepared by Gonzalezmartin et al. (2011) showed a certain sustained-release effect with 65.4% of drug release within 6 hours at pH 7.4. Its acaricidal activity against Trypanosoma cruzi was significantly increased compared to the solution. Similarly, Labhasetwar and Dorle, (1990) prepared gelatin, albumin, gluteraldehyde and polyacrylamide nanoparticles with varying from 85 to 1200 nm in size demonstrated *in vitro* sustained release. Compared with other nanoparticles, polymer nanoparticles hold better stability (Lala & Basu, 2004). In addition, some natural polymer are cost-effective and have no obvious side effects, which make it advantageous. For some polymeric nanoparticles, chemical reactions and organic reagents can be avoided to let the nanoparticles more safer. For example, the ionotropic gelation does not require the introduction of a chemical group into a methyl group (Vaezifar et al., 2013), which showed good safety with stronger effect against toxoplasma (Hagras et al., 2019) and enhanced antibacterial activity (Etewa et al., 2018).

## 4. Influences of nanoparticle properties on the activity of their loaded antiparasitic drugs

### 4.1. Size

The nanoparticle size plays an important role in its transport behavior and distribution *in vivo*. The nanoparticles with different sizes might be distributed differently in the body and thus have different inhibitory effects on parasites. Liu et al. (1992) found that 60% radiolabeled liposomes with a size of 100–200 nm were distributed in the blood 4 hours post-dose, while only 20% liposomes over 250 nm or less than 50 nm was distributed in the blood after injection of radiolabeled liposomes with the sizes of 30–400 nm to mice. The nifurtimox nanoparticles prepared by Gonzalezmartin et al. (2011) has a particle size of less than 200 nm, which greatly prolongs the blood circulation time and enhances the activity of the Trypanosoma cruzi. Nearly 60% of the 50 nm nifurtimox nanoparticles were distributed in the liver, while only 25% of 100 nm and over 250 nm particles are accessible to the liver. This phenomenon might be attributed to that the size of liposome with 50 nm is smaller than the discontinuous window-like structure of the liver endothelial cells, which is beneficial to its penetration into the liver, thereby improving its distribution in the liver. It is realized that spleen is another main target tissue for nanoparticles. It is reported that the distribution of nanoparticles in the spleen was significant as the increase in the size of the nanoparticles. The liposomes of about 100 nm showed the least spleen distribution, while 40%–50% of administration liposomes with a size of approximately 400 nm were distributed in the spleen. Besides, some studies have found that the smaller the particles, the easier it is to excrete by the urinary system (Chen et al., 2013). Therefore, it is a promising way to enhance antiparasitic drug residence and target distribution where the parasites resided via controlling the size of nanoparticles.

### 4.2. Shape

The shape of the nanoparticles has a positive influence on the macrophage phagocytosis, diffusion rate and distribution in the body, and thus affecting the pharmacokinetics of its payload. Chen et al. found that the distribution of disc-shaped particles in the lungs and heart was significantly higher than that of other shapes, while cylindrical nanoparticles were significantly higher in the liver than other shapes (Chen et al., 2013). Geng et al. found that it is difficult for macrophages to devour rod-shaped nanoparticles *in vitro* (Geng & Discher, 2005). After an intravenous injection to mice, the rod-shaped nanoparticles showed extended biological half-life up to 5 d. Other researchers also found that the rod-shaped nanoparticles were less likely to be phagocytosed into cells than granular nanoparticles via comparing phagocytosis of nanoparticles of different shapes *in vitro* cell culture methods (Chithrani & Chan, 2007). It is found that the recognized velocity of different shape of nanoparticles when the volume is in the range of 0.075–0.69 μm^3^ by phagocytic cells was in the following order: rod-shaped > oblate ellipsoid > spherical, while the phagocytosis rate is oblate ellipsoid > spherical > rod-like. Therefore, the nanoparticles can be made into a specific shape to control their cellular entrance ability and thereby increasing the insect repellent efficacy of drugs. It is more important for treating intracellular parasitic infections, e.g. spherical amphotericin nanoparticles for Leishmania.

### 4.3. Surface charge

The distribution and metabolism of nanoparticles are also influenced by their surface charges. Levchenko et al. (2002) found that the clearance rate of nanoparticles with negative surface charge in mice was significantly higher than that the nanoparticle with a neutral surface charge. For example, the fenbendazole liposomes with a neutral surface charge have a longer circulation time in the blood and easily enters the brain through the blood-brain barrier (BBB), and thus exhibit a stronger effect on the bow worm in the brain. Similarly, the distribution of nanoparticles with negative surface charge in the liver was also significantly higher than that of the nanoparticles with a neutral surface charge. These results indicated that charged nanoparticles are more likely to be engulfed by macrophages in the liver. Reportedly, nanoparticles with positive surface charge are easily agglomerated by binding to negative potential serum proteins after entering the blood (ZHANG et al., 2005). The agglomerated nanoparticles obtain large particle size and then are prone to transient blockage in the capillary of the lung tissue. After the dissociation of the nanoparticles from bounded serum proteins, the nanoparticles will be redistributed to tissues. These processes lead to its longer clearance time compared to the nanoparticles with a negative surface charge. It is also reported that the surface negative charge liposomes easily accumulate in the muscles, which contributes to the treatment of toxocariasis in the muscles (Velebný et al., 2000).

### 4.4. Surface hydrophobicity

The surface hydrophilicity (hydrophobicity) of nanoparticle also shows a great influence on its kinetics *in vivo,* mainly via changing its protein binding extents and rate of the nanoparticles *in vivo*. Basing on these, we can modify the surface hydrophilicity (hydrophobicity) of the nanoparticles to achieve the expected distribution and kinetics. Studies have shown that polyethylene (PEG) modification can improve the hydrophilicity of nanoparticles and thus reduce the protein binding rate. Also, PEG modification can reduce or eliminate the surface charge of nanoparticles. Therefore, PEG modification can significantly prolong the biological half-life and residence time of nanoparticles (Pensel et al., 2015; Kumar et al., 2017; Fülöp et al., 2018). Meier et al. reported that the residence time and bioavailability PEG-modified liposomes were 6 and 36 times larger than those of PEG-free nanoliposomes, respectively (Kumar et al., 2017). In the treatment of parasitic diseases, it often needs prolonged or repeated usage of drugs. Therefore, surface hydrophilicity modification of nanoparticles is essential to obtain satisfactory sustained-release performance and thus further improve the therapeutic effects of antiparasitic drugs.

## 5. Transports of nanoparticles *in vivo*

**Figure 1.**
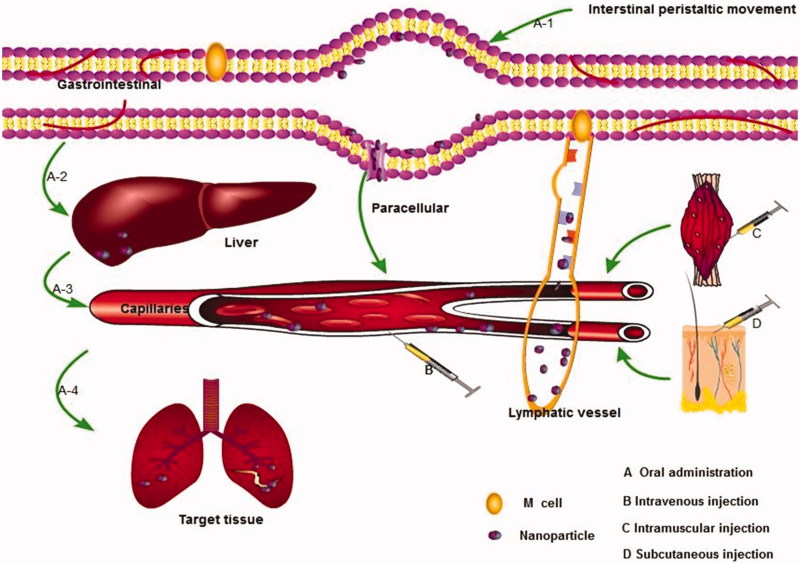
The transport process of nanoparticle *in vivo*.

The transport kinetics of nanoparticles *in vivo* is a complex process after entering the body, which mainly includes transporting in blood vessels, penetrating the vessel wall into the interstitial space, transporting tissue gaps, and entering cells where parasites reside (Figure 1). The nanoparticles could achieve effective absorption, sustained-release, and targeting to the parasitic resided sites via the above-mentioned transport process and thus obtain enhanced therapeutic effects. However, there are few studies on the transport of antiparasitic nanoparticles *in vivo* up to now. In this section, the *in vivo* behavior of nanoparticles mainly based on the antitumor drugs and antibacterial drug-loaded nanoparticles will be summarized to guide the design of nanoparticle delivery systems for antiparasitic drugs.

### 5.1. Absorption of nanoparticles

Effective transport across membrane barriers is necessary for the absorption of drugs. The ultra-small size and huge surface area of nanoparticles are easy to adsorb on the tissue and cell surface, which will result in high concentration and long residence of nanoparticles and their payload at the medication site. The huge adhesive force and small size contribute to their complete absorption. It is reported that albendazole-loaded SLNs with large surface due to their small size of about 157.8 ± 2.82 nm held strong adhesion to the epithelial cells of the gastrointestinal mucosa and thus ensured that the relative bioavailability of their loaded albendazole was doubled compared to the common suspensions (Liu et al., 2013). The nanoparticles might penetrate the biofilm barrier through transcellular and paracellular transport. For oral and injection administration, the lymphatic pathway is also important for their absorption and sustained release performance *in vivo* (Desai et al., 1996; Conner & Schmid, 2003).

#### 5.1.1. Transcellular transport absorption

**Figure 2.**
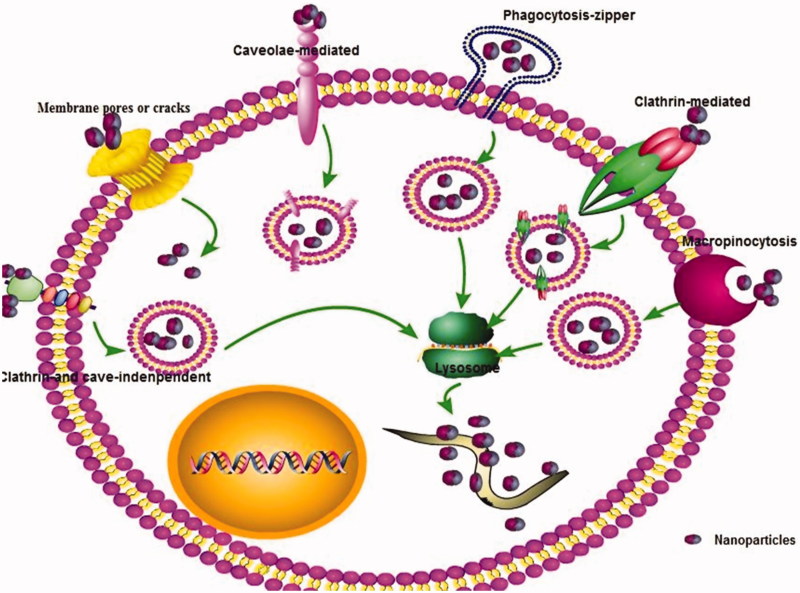
The cellular uptake pathways of nanoparticle.

Currently, it is generally believed that the transcellular pathways are one of the major absorption pathways for nanoparticles. Endocytosis is the main pathways for transcellular transport of nanoparticles across the organism membrane barriers. The cells firstly recognize nanoparticles via a selection of the receptors of the cell surface after opsonin. Next, the plasma membrane was induced to form small vesicles, and then the small vesicles formed by invagination are separated from the plasma membrane into cells, fused with lysosomes, enzymatically hydrolyzed or hydrolyzed to release drugs. This endocytosis could be further divided into phagocytosis and pinocytosis, which is mainly determined by the properties of nanoparticles and target cells. Most of the nanocarriers are aqueous dispersions or converted into aqueous dispersions *in vivo*. The liquid dispersions penetrate the biological membrane barrier mainly through pinocytosis (Conner & Schmid, 2003; Mayor & Pagano, 2007). Pinocytosis is classified as ‘caveolae-mediated endocytosis (CvME), clathrin- and caveolae-independent endocytosis, clathrin-mediated endocytosis (CME), and micropinocytosis’ (Figure 2). Among them, CME is the one of the most important pathways of most nanoparticles to enter cells (Santiwarangkool et al., 2019; Wu et al., 2019). For example, Santiwarangkool et al. (2019) found that GALA-modified liposomes were entered the lung endothelial cells mainly via a CME. The uptake of asenapine maleate-SLNs across the Caco-2 cell line was mainly via clathrin-mediated endocytosis transport with time and energy-dependent way (Patel et al., 2019). The nanoparticles with different sizes may enter cells in different ways. It should be noted that caveolin-mediated pinocytosis occurs membrane region, which is formed by particle of about 60 nm and pin necked bottle membrane functional area with a specific diameter of 50–100 nm on the cell membrane surface covered by a caveolin to form a vesicle into the cell. The caveolin-mediated pathway does not fuze with lysosomes, which can avoid ligand degradation and transport to the intracellular or extracellular domain in a functionally active state (Conner & Schmid, 2003). Because of the quite diverse of these nanoparticles, understanding the different mechanisms and ways that help in the regulation of nanoparticles internalization is important for development of antiparasitic drug-loaded nanoparticle. The transcellular transport process and types and their influences have been revised in our previous review (Xie et al., 2014).

#### 5.1.2. Paracellular transport absorption

Physiologically, the adjacent endothelial cells are filled with fluid. The membrane of the top side of cells is connected to form a tight junction, which hinders the paracellular transport of drugs. Common absorption enhancers can improve the cell connective membrane permeability and thus enhance the absorption of drugs. By changing the surface properties of nanoparticles or the dispersion medium, the cytoskeleton and tight junction-related protein-membrane distribution of membrane can be changed. The tight junction can be opened via surface modifications to improve the intercellular transport and absorption of nanoparticles and their bound drugs. Studies have shown that unsaturated fatty acid or some surfactants such as Tween-80 (T-80), the commonly used excipients of nanoparticles, can temporarily open or widen the tight junction channels between epithelial cells. When chitosan swells, they can mediate the structural reorganization of tight junction protein of epithelial cell via ionic interactions and increases paracellular transport capacity. It is reported that the intercellular space and permeability of endothelial cells was significantly increased when treated with T-80 modified nanoparticles (He et al., 2005).

#### 5.1.3. Lymphatic transport absorption

The Peyer’s patches (PPs) of the gastrointestinal tract is the most important way except the trans-intestinal epithelial absorption to absorb nanoparticles after oral administration. The M cells on PPs as functional cells can open the ideal channel for the intestinal mucosal barrier, which is the main non-receptor transport pathway of nanoparticles. After phagocytosis, the nanoparticles are transported to the M cells by cystic transport and then enter the blood circulation from the lymphatic circulation in a free state or a phagocytic state. In this pathway, the nanoparticles are absorbed into the blood in a complete structure, which can effectively protect encapsulated drugs from gastrointestinal degradation and first-pass metabolism. This way has important clinical significance for the absorption of unstable drugs and drugs with strong first-pass effect, such as praziquantel and benzimidazole (Desai et al., 1996; Kreuter, 2001). PPs absorption is a unique pathway for the uptake of nanoparticles, and the degree of absorption is related to particle size to an extent. Amongst a certain range, the degree of lymphatic transport is inversely proportional to the nanoparticle size. The smaller the nanoparticle size, the greater the degree of lymphatic absorption, while the lymphatic absorption is unchanged or even could not be absorbed when the nanoparticle size was increased to a certain extent (He et al., 2005; Kreuter, 2001). Also, many nanoparticles could pass through the lymph by oral, subcutaneous, and intramuscular administration. The circulation time in body can last for about 24 h to a week since that lymph fluid flows slowly and the lymphatic system acts as a large reservoir of drug storage.

#### 5.1.4. Direct drug molecular absorption

The nanoparticles payload also could be transported in the form of molecular state. For example, due to its nanometer size, nanoparticles adhere to the gastrointestinal mucosa after oral administration, and its huge surface area makes the drug more dissolution, according to the difference in concentration inside and outside the membrane, it can be directly transferred into the blood circulation in the form of molecules by active transport or passive diffusion (Varshosaz et al., 2018). When the particles reach the nanoparticles level, the total surface area and curvature of the drug will be increased, which contribute to enhancing the dissolution rate of drug, especially for some insoluble antiparasitic drugs. According to the Kelvin formula, the solubility of the drug will be significantly improved when the size of the particle is reduced in the range of nanosized range. The increase in solubility and dissolution will undoubtedly enhance molecular drug absorption.

### 5.2. Distribution of nanoparticles

The nanoparticles are to achieve distribution via blood circulation. The nanoparticles hold a selective distribution *in vivo* due to their unique particle characteristics and surface properties. The nanoparticles loaded with antiparasitic drugs can passively target to the infection site via the recognition and transport by ‘the enhanced permeation and retention (EPR) effect and the mononuclear phagocytic system (MPS)’ because of the locally improved permeability of microvascular capillary and drainage of impaired lymphatic system by inflammatory effects. Therefore, nanoparticles are easily distributed in liver and kidney tissues with being abundant in the reticuloendothelial system after entering the blood. The distribution of nanoparticles could be adjusted via change their shape, size, surface morphology, constituent, and administration routes. The passive and active distribution will target the parasitic infection site and reduce its toxicity to non-target organs.

The particle size can significantly influence their distribution and play an essential role in nanoparticle design. It is realized that nanoparticles with a smaller size have longer circulation times in the body (Dufort et al., 2012). This is often found if nanoparticles >100 nm are compared with nanoparticles <100 nm. Generally, nanocarriers of 100–200 nm are easily removed from the blood by the reticuloendothelial system. The larger nanoparticles are more quickly phagocytized by the reticuloendothelial system and swifter eliminated from the blood to reach the liver and spleen tissues with rich reticular endothelial. However, the trend of the longer circulation times of small-sized nanoparticles is not always noticeable when the diameters of nanoparticles range from 10 to 100 nm. As shown in PEGylated polyacrylate nanoparticles when its size changed from 20 to 60 nm, the systemic clearance rate and liver accumulation were decreased significantly (Yang et al., 2009). Homoplastically, 25 nm micelles displayed much shorter elimination half-life than 60 nm polymer micelles (Lee et al., 2010). This observation was hypothesized by some researchers regarding to the clearance of the smaller micelles by hepatobiliary excretion since about 70% of the liver fenestrations of mouse are smaller than 100 nm. Therefore, nanoparticles below 100 nm can easily enter the liver parenchyma cells (Jong et al., 2008). It was found that the longest elimination half-life of PEGylated gold nanoparticles within 10–100 nm was accomplished by compromising between the sizes and surface PEG chain length of nanoparticles, resulting in 60 nm particles (Perrault et al., 2009). It is realized that the nanoparticles less than 50 nm can penetrate the capillary endothelium of the liver, pancreas, intestine, stomach, or pass through the lymph to the spleen and bone marrow cells. Nanocarriers below 10 nm are easily to slowly accumulate in the bone marrow (Kreuter et al., 2002; Liu et al., 2010). Oussoren et al. (1997) reported that 76% of 40 nm liposomes were detected in the lymphatic system after intramuscular injection, while the larger has remained at the injection site. It is reported that the small particles were directly absorbed into the lymph node tissue, and the large particles are absorbed by physical filtration when liposomes in size range of 48–720 nm were administered intraperitoneally. It is swallowed by macrophages for lymphatic targeting when they were transported through the lymphatic vessels (Nishioka & Yoshino, 2001). Generally, reducing in the nanoparticle size in a certain range is often considered to be one of the possible effective methods to extend the circulation time of nanoparticles, because smaller nanoparticles are effective in avoiding RES phagocytosis *in vivo*. For the treatment of parasitic brain infections, the pronged circulation of nanoparticles and their payload via adjusting the size should attract attention. However, Hirsjärvi et al. found that the 25–100 nm nanocarriers showing constant distribution rates in different tissues and there is no strong connection between the size of nanoparticle and the distribution profiles. They also showed that biodistribution is similar to the complement activation and macrophage phagocytosis *in vitro* and no apparent differences between the nanoparticle types (lipid nanocapsules versus lipid nanoemulsions) (Hirsjärvi et al., 2013).

Studies have found that the surface charge and properties of nanoparticles can directly affect its binding to proteins resulting in their quick distribution. Under the constant particle size and hydrophobicity, the surface positively and negatively charged nanoparticles can increase the amount of plasma protein bind as the surface charge density was increased. Nanoparticles with a positive charge are preferentially bound with proteins of Isoelectric point (PI) <5. 5 (such as albumin), and those with negatively charged or acidic groups are preferentially bound with proteins of PI >5. 5 (such as lgG). The surface charged particles are effortlessly removed from the body, while the nanoparticles without surface charge are more suitable for long circulation. Therefore, it is will be an effective way to modify antiparasitic drug-loaded nanoparticles with nonionic surfactants. It was also found that albumin and lgG were preferentially adsorbed on nanoparticles with basic groups or weak acid groups on the surface (Aggarwal et al., 2009). Literature showed that PEG-modified nanoparticles bind only a small amount of protein and can circulate in the blood for a longer time (Meier et al., 2003; Womack, 2006; Boyd, 2007) compared to the unmodified nanoparticles (Aggarwal et al., 2009). It is also found that the possibility of being swallowed is reduced as the surfactant layer thickens of nanoparticles was increased. The surface layer thickness of more than 10 nm can effectively exert spatial steric hindrance and reduce their recognition. Interestingly, the tween-modified nanoparticles can be selectively targeted to the brain via the blood-brain barrier, which will be beneficial for the treatment of parasitic brain infections.

### 5.3. Elimination

#### 5.3.1. Metabolism

The metabolism of nanoparticles includes the process of throughout changing their physicochemical properties. Nanoparticles are transported to the liver through the portal vein and metabolized once that they are absorbed by the gastrointestinal tract. The endocytosis of nanoparticles by the reticuloendothelial system can accelerate the metabolism of nanoparticles. Some nanoparticles, such as amphotericin B liposome (Rathore et al., 2011) and artemisinin liposome (Dwivedi et al., 2014), can fuze with cell membranes and then enter cells for the treatment of intracellular parasites. After entering the cell, the nanoparticle can be hydrolyzed by the lysosomes of the cell, releasing the drug to exert its efficacy. See et al. showed that breastfed animal cells could take up nanoparticles by endocytosis, and the surface biomolecules of the nanoparticles can be decomposed by cathepsin L (Sée et al., 2009). The metabolism of nanoparticles mainly depends on their composition. The metabolism of nanoparticles prepared by synthetic polymers and natural polymers is mainly up to the degradation of skeleton polymers (Asthana et al., 2015). The SLNs with different fatty acid as a lipid matrix obtained different targeting and sustained release by adjusting the metabolic velocity of the drug in liver (Kayser, 2001; Dwivedi et al., 2014). The metabolism of the nanoparticles in the liver is also influenced by the properties of nanoparticles, which can affect the bioavailability of the nanoparticle payload. To avoid the first-pass effect as much as possible and prolong the blood circulation time, the properties of nanoparticle surfaces could be optimized (Dwivedi et al., 2014). It is also an effective way to change the *in vivo* circulation time by surface modification of the nanoparticles with different polymers. The modification of PEG can avoid the recognition of opsonin and prevent it from being taken up by the reticuloendothelial system and thus obtained a long-circulating effect (Zhang et al., 2002). Other substances, such as methotrexate, polyethyleneimine, and dextran, are also used to modify the nanoparticle surface and change their charge (Kango et al., 2013; Sukhanova et al., 2018), thereby reducing the metabolism and achieving the role of targeting.

#### 5.3.2. Excretion

Besides the recognition by MPS and metabolism of a liver metabolic enzyme, the difficulties for the prolong residence of nanoparticles in the body have kidney glomerular excretion and hepatic sinusoidal capillary capture. It is realized that the excretion pathway of nanoparticles is related to the size of nanoparticles. He et al. (2011) found that smaller nanoparticles (80 ∼ 120 nm) can be excreted through the kidney, and the metabolites of larger particles are mainly through hepatobiliary excretion. Protein binding and phagocytosis of RES play a key role in the removal of foreign materials from plasma to the liver. Simpson et al. found that different sizes of glutathione-coated gold nanoparticles after intravenous injection into the rat is completely excreted through the urine (Simpson et al., 2013; Sancey et al., 2015). Sancey et al. (2015) found most of the 20–200 nm Gadolinium-Based activation guiding of irradiation by X-rayA (GuIX) nanoparticles were partially eliminated by the liver after intravenous administration to rats, while the small diameter of the nanoparticles showed rapid kidney accumulation and renal clearance. The surface charge could affect excretion rates. For example, it is reported that the higher-charged nanoparticles (+34.4 mV) were rapidly transported from the liver into the gastrointestinal tract and subsequently excreted through the feces, while the less charged nanoparticles (–17.6 mV at pH 7.4) remain isolated in the liver (Souris et al., 2010). The routes of administration also have important effects on nanoparticle distribution and excretion (Chen et al., 2017). Our previous work demonstrated that hydrogenated castor oil-solid lipid nanoparticles extended the MRT of praziquantel from 6.6, 7.6, and 8.2 to 151.6, 95.9, and 48.2 h after subcutaneous, oral and intramuscular routs, respectively (Xie et al., 2010).

## 6. Challenges and prospects

Nanocarriers are effective and prospect as potential delivery systems for antiparasitic drugs. Currently, the sustained release, enhanced absorption, and intracellular delivery of nanoparticles are hot issues for antiparasitic drug delivery. Although there are no antiparasitic drug nanoparticle formulations on the market, dozens of nanoparticle formulation are undergoing formulation designs, preclinical studies, or clinical trials. Most studies have shown that the nanoparticles would be promising in the treatment of parasitic disease. Currently, those goals of into nanoparticle commercialization for antiparasitic drug delivery are still very far from completion, and there is still a series of challenges to be solved for their coming into the market, although it has bright opportunities. In the future, bioactive macromolecular antiparasitic drugs will be an important development direction in the future, while these drugs have unsatisfactory stability and absorption. The nanocarriers will be a satisfactory vehicle for bioactive macromolecular antiparasitic drugs and an inevitable trend in the development of pharmaceutical dosage forms.

It is well realized that nanoparticles can be quickly cleared from the blood by phagocytes, which is beneficial for the treatment of the parasites live in the liver, spleen, and lymphatic system. For the treatment of parasites resided outside of MPS, the ideal sustained release is very necessary. Achieving the expected sustained release is one of the significant challenges for different parasitic disease treatments. One possible way is to adjust the elimination half-life of nanosystems via controlling passive target and endocytosis of MPS in resident sites. It is generally realized that small, hydrophilic, and neutral nanocarriers are not easily recognizable by MPS by hindering the adsorption of opsonin on nanoparticle surface. Therefore, controlling the constituent and properties of nanoparticles will be a possible way to control their circulation. To avoid phagocytosis by macrophages, the nanoparticles should be modified on their surface. In the past, PEG-based synthetic polymers were often used to alter their surface properties. Current research demonstrated that some hydrophilic natural and synthetic polymers such as polysaccharides show more practical effects. The surface-modified with functional groups and materials should be strengthened. Except MPS recognition, the hinder for the sustained release of nanosystems contain kidney excretion and biliary excretion. According to the preliminary research of size on the excretion, controlling nanoparticles into the range between 100 and 200 nm could be possible to enhance their circulation time *in vivo*.

As summarized previously, nanocarriers hold the advantages of delivering their laden drugs into cells and organelles using smart cellular uptake and intracellular transport pathways. Recently, increasing researches demonstrate that the physical and chemical properties of nanoparticle could influence their interaction with the cellular surface and the subsequent endosomal properties, thus mastering the cellular uptake and intracellular transport of nanoparticles and their payload release (Xie et al., 2014). The new understanding how the nanoparticles control their intracellular delivery via themselves properties is not fully studied. There are few reports about influences of antiparasitic drug-loaded nanoparticles properties on their intracellular distribution. Future studies should focus on exploring the decisive factors in controlling the cellular entry and intracellular destiny of nanoparticles to guide the design of the nanoparticles towards target cells and organelle where parasite resides, and to control their intracellular release.

To implement the final application of nanoparticles, there is not only a necessary to ensure that nanoparticles have the enhanced absorption, sustained release, target effects and intracellular delivery at adequate concentrations for an expected period to obtain satisfactory therapeutic results, but also nanoparticles should be safe, inexpensive, and easy and reproducible manufacture in large scale. It is a pity that the nanoparticles seldom ultimately achieve previous requirements. Currently, the safety of nanoparticles run short of systemic study and attention. The core concept of carefully designed nanoparticles is to use fewer drugs to obtain more potent effects. Although, studies reported that most nanoparticles are safe and even can reduce the side effects of drugs compared to traditional drug formulations. However, studies have shown that some nanoparticles with highly intensive charged zeta potential have specific toxicity. Discovery and synthesis of the novel biocompatible and biodegradable nanomaterials with nontoxicity should be paying more attention. The chemical structure of the nanoparticle’s materials and their metabolites should hold excellent physiological and biological compatibility, nontoxic and non-immunogenic toxicity, and can be completed exclusion of the body in a reasonable time in the development of antiparasitic drug-loaded nanoparticles. After preparation into nanoparticles, its safety should be systematically evaluated using the proper biosafety evaluation system at molecule, cell, tissue, organ, and organism levels. Also, nanoparticles might have specific effects on the water, soil, etc. due to their unique properties after nanocrystallization of bulk materials, which may threaten animals and plants and even humans in the natural environment. The environmental safety of nanoparticles should be attracted more attention. The risk assessment of nanoparticles on the environment and human should be evaluated based on some suitable models and then establish related management regulations. The industrial production is currently a big challenge for the clinical application process of nanoparticles, especially for the polymeric nanoparticles. The preparation technology of many kinds of nanoparticles is still in the laboratory preparation research stage. It will take a lot of time and effort to drive the nanoparticle delivery system into the commercial and large-scale production stage. Currently, our groups have successfully established the widespread production techniques for SLNs suspensions and nanocrystal nanosuspensions, which will be beneficial for their industrialization and application.

In summary, more focus must be made in the systematic investigation and research on the effectiveness and conclusive factors of nanoparticles for determining their special locus targeting of antiparasitic drugs, enabling the designed nanoparticles with hopeful pharmacokinetic and pharmacodynamic properties. The fine but rich challengeable aims lie in creating smart nanocarriers simultaneously possessing several functions to ensure satisfactory absorption, long-circulation time and targeting, and low or nontoxicity. Furthermore, the research method and powerful technologies should be increasingly and significantly improved to accurately study the *in vivo* fates and pharmacodynamics of nanoparticles. With constant efforts, the treatment effects by nanoparticles-loaded antiparasitic drugs will continue to be enhanced and have an infinite future for the efficient therapy of parasitic disease.
